# Parental psychological control and smartphone addiction in Chinese college students: the moderated mediation roles of self-control and achievement motivation

**DOI:** 10.3389/fpsyg.2026.1873493

**Published:** 2026-08-19

**Authors:** Yang Bai, Yuan Peng, Yuanyuan Chen, Hua Wang

**Affiliations:** 1 Teacher’s College, Xi’an University, Xi’an, China; 2 Department of Psychology, Shaanxi University of Technology, Hanzhong, China

**Keywords:** achievement motivation, parental psychological control, self-control, smartphone addiction, the moderated mediation model

## Abstract

Although smartphones provide numerous benefits in daily life, evidence indicates that smartphone addiction has many potential risks. The effect of parental psychological control on smartphone addiction has been well established; however, little is known about the potential mechanisms underlying this relationship. Thus, we tested a moderated mediation model in which self-control was hypothesized to mediate the association between parental psychological control and smartphone addiction among college students, while achievement motivation was hypothesized to moderate the indirect pathway. A sample of 730 Chinese college students (*M*_*ag*e_ = 19.22 years, *SD* = 0.79) anonymously completed questionnaires assessing parental psychological control, self-control, achievement motivation, including approach motivation and avoidance motivation, and smartphone addiction. The results indicated that (1) self-control mediated the association between parental psychological control and smartphone addiction and (2) the indirect pathway was moderated by approach motivation instead of avoidance motivation. Specifically, the association between lower self-control and smartphone addiction depended on the level of approach motivation. Higher self-control was related to lower levels of smartphone addiction, regardless of the level of approach motivation. These findings highlight the complex interplay between family environmental factors and individual characteristics in understanding smartphone addiction among college students.

## 1 Introduction

In the past few decades, smartphone adoption has steadily increased worldwide due to their accessibility and functional diversity. Although smartphones provide considerable benefits in daily life, excessive and uncontrolled use may lead to smartphone addiction (Billieux, 2012; Volkmer and Lermer, 2019). Smartphone addiction, defined as excessive and uncontrolled smartphone use, is broadly viewed as a subset of behavioral addiction (Billieux, 2012; Liu et al., 2020; Griffiths, 2017). Smartphone addiction has been a worldwide phenomenon and is increasingly common among college students (Jiang and Zhao, 2016; Lepp et al., 2014). Previous studies have suggested that smartphone addiction is associated with maladaptive outcomes, such as physical and mental health problems, interpersonal impairment, and academic failure (Chen et al., 2016; Liu and Wang, 2011).

To better understand the factors influencing the prevalence of smartphone addiction, a number of studies have explored potential risk factors involved in smartphone addiction (Busch and McCarthy, 2021), particularly by separately investigating the associations between environmental factors (e.g., family functioning, parental marital conflict, and parental control) and individual variables (e.g., personality traits, impulsivity, shyness, introversion, social withdrawal, depression, and poor self-control) with smartphone addiction (e.g., Busch and McCarthy, 2021; Darcin et al., 2016; Jiang and Zhao, 2016; Lian et al., 2016).

However, few studies have examined the combined effects of environmental factors, such as parental psychological control, and individual variables such as self-control, on smartphone addiction among college students based on the person–environment interaction perspective (e.g., Cicchetti, 2008). According to the person–environment interaction perspective, maladaptive behaviors emerge from the dynamic interplay between environmental experiences and individual characteristics rather than from either factor alone (Cicchetti, 2008). Therefore, parental psychological control may influence smartphone addiction not only directly but also indirectly through individual psychological characteristics. Within the person–environment interaction perspective, motivational characteristics may further shape how environmental influences are translated into behavioral outcomes. In addition, according to the dual-motive model (Fujita, 2011), self-control and motivation may interactively influence an individual’s behavior. Thus, we investigated the combined effects of parental psychological control, self-control, and achievement motivation on smartphone addiction among college students. Specifically, the present study examined whether achievement motivation moderates the mediation effect of self-control in the association between parental psychological control and smartphone addiction among college students.

### 1.1 Parental psychological control and smartphone addiction

As the most essential and persistent microsystem, the family environment exerts a long-lasting effect on an individual’s development. Parental psychological control, which is one of the core variables within the family environment, plays a key role in children’s psychosocial functioning (Baumrind, 1991). Parental psychological control refers to parents’ attempts to control children’s psychological and emotional development by expressing disappointment, inducing guilt, withdrawing love, and asserting authority (Barber, 1996).

In the Chinese cultural context, parental psychological control may exert particularly strong developmental influences. Rooted in Confucian traditions and collectivist values, Chinese parents often regard greater parental control as an expression of care and concern (Chao, 1994; Wang et al., 2025). Previous studies have shown that psychologically controlling parenting is associated with higher levels of both internalizing and externalizing problems among Chinese youth (Feng et al., 2025; He et al., 2023). Although parents may intend to facilitate their children’s successful adaptation, psychologically controlling parenting can undermine the development of autonomy and identity, thereby hindering long-term psychosocial adjustment and leading to negative consequences that may be difficult to reverse (Charalampous et al., 2018; Loeb et al., 2021; Yaffe, 2021; He et al., 2023).

Although several studies have shown that the salience of parental influences declines as individuals develop into young adults (Windle, 2000; Wood et al., 2001), there is considerable research suggesting that parents continue to influence their children even after they move to college, across domains such as financial functioning, academic functioning, emotional, social, and behavioral functioning (Abar and Turrisi, 2008; Fass and Tubman, 2002). Research has found a strong association between parental psychological control and problem behaviors among children and adolescents (Lee et al., 2016). However, only a few studies have examined the association between parental psychological control and smartphone addiction among college students (e.g., Lian et al., 2016).

Thus, the first aim of the present study was to examine the association between parental psychological control and college students’ smartphone addiction. From the perspective of person–environment interaction, parental psychological control represents a distal environmental risk factor that may influence smartphone addiction both directly and indirectly through individual psychological mechanisms. Therefore, the following hypothesis was proposed:

*Hypothesis 1*: Parental psychological control is positively associated with college students’ smartphone addiction.

### 1.2 Self-control as a mediator

The component model of behavioral addiction (Griffiths, 2005) proposes that the environmental factors and individual variables jointly contribute to behavioral addiction, including smartphone addiction (Bae, 2015). Beyond the direct effect, parental psychological control might have an indirect effect on college students’ smartphone addiction through psychological pathways. The study of the mediation pathway is essential to inform a better understanding of how parental psychological control is associated with smartphone addiction and for designing effective interventions to prevent or reduce smartphone addiction. However, the mediating mechanism through which parental psychological control is linked to college students’ smartphone addiction has not been thoroughly examined. The present study considered self-control as a potential mediator between parental psychological control and college students’ smartphone addiction. Within the person–environment interaction perspective, self-control represents a key personal characteristic through which environmental experiences influence behavioral outcomes. Self-control enables individuals to control their thoughts and actions when the ongoing task is unwanted or inappropriate and inconsistent with their long-term goals (Tangney et al., 2004). It plays an important role in multiple life domains, including health, academic achievement, and vocational functioning (Pesce et al., 2020), and is a crucial, but often overlooked, ingredient for success in most treatment programs.

Parents play essential roles in children’s behavioral development, especially in the development of their self-control (Hay, 2001; van Prooijen et al., 2018; Liu et al., 2025). According to the self-determination theory (Ryan and Deci, 2017), parental psychological control could undermine individuals’ autonomy and self-control by causing them to believe that events in their lives are uncontrollable and unpredictable (Chorpita and Barlow, 1998). That is, children who experience parental love withdrawal and manipulative control may lack opportunities to learn emotion and behavior regulation and fail to develop a sense of themselves as competent individuals capable of controlling events in their lives (Li et al., 2015; Nanda et al., 2012; Xing et al., 2017). The majority of studies have shown that parental psychological control was negatively associated with children’s self-control (Crosswhite and Kerpelman, 2012; Li et al., 2014; Nie et al., 2016). Based on a developmental neuroscience hypothesis (Casey et al., 2008), the full maturation of the brain’s frontal cortex, which underlies self-control, persists into the third decade of life. Longitudinal studies have also provided evidence that the development of self-control persists into adulthood (Burt et al., 2014; Romer et al., 2010). Thus, although college students no longer rely on their parents to the same extent as they did during childhood, parental psychological control may still influence the development of their self-control.

On the other hand, the self-control theory (Gottfredson and Hirschi, 1990) proposes that individuals with poor self-control are prone to act more impulsively, are prone to engage in risk-taking, and are sensitive to immediate gratification and rewards (Niemz et al., 2005). Therefore, individuals with poor self-control may have difficulty in making right decisions and acting in a manner consistent with their long-term goals and are likely to indulge in tempting, immediately gratifying rewards and impulses in the virtual world (Fujita, 2011). Previous studies have suggested that low self-control is a primary factor underlying many problem behaviors, including violence, risk-taking, and Internet addiction (Özdemir et al., 2014). Individuals with low self-control are more likely to overuse smartphones (Akn et al., 2015; van Deursen et al., 2015). In contrast, effective self-control could be a protective factor that enables individuals to prioritize long-term goals over temptations and immediate satisfaction derived from the virtual world. Evidence also shows that higher self-control is negatively associated with smartphone addiction (Jiang and Zhao, 2016). Although evidence has indicated that low self-control predicts college students’ smartphone addiction, limited research has examined its link with smartphone addiction when considering the effect of parental psychological control. Thus, the following hypothesis was proposed:

*Hypothesis 2*: Self-control mediates the relation of parental psychological control to college students’ smartphone addiction.

### 1.3 Achievement motivation as a moderator

The mediation effect focuses on the “process” and “commonness” underlying variable relations. However, it cannot answer the question of under what circumstances the effects of parental psychological control and self-control on smartphone addiction are stronger or weaker. In addition, although a majority of studies have suggested a mediation role of self-control on addictive behaviors, some inconsistent findings have also been reported. For example, Özdemir et al. (2014) found that the mediation effect of self-control between depression and Internet addiction was not significant. Accordingly, the indirect relationship between parental psychological control, self-control, and smartphone addiction may be moderated by certain factors. According to the dual-motive model (Fujita, 2011), the self-control dilemma is fundamentally a dual-motive conflict that reflects the conflict between a concrete, proximal reward and an abstract, remote goal. Successful resolution of the self-control problem requires individuals to act consistently with the motivation directed toward the more abstract and distal goal. If individuals are more strongly motivated to pursue abstract and distal goals, they are more likely to exert effective self-control (Muraven et al., 2006). Therefore, achievement motivation is more likely to influence smartphone addiction by shaping the effectiveness of self-control rather than by exerting a direct effect on addictive behavior.

As a form of social motivation, achievement motivation is an important part of non-intellectual characteristics, which are also an essential determinant of human behavior (Elliot and Covington, 2001). Recent evidence from Chinese college students suggests a generational decline in approach motivation and an increase in avoidance motivation (Yang et al., 2026), highlighting the increasing importance of achievement motivation in young adults’ behavioral adjustment. According to the hierarchical model of approach-avoidance motivation (Elliot and Church, 1997), achievement motivation refers to the internal driving force that motivates individuals to achieve success, and generally it can be divided into two forms: approach motivation and avoidance motivation. Approach motivation and avoidance motivation have several fundamental distinctions.

First, approach motivation and avoidance motivation differ in their valence function and behavioral directions. Individuals with approach motivation are likely to regard tasks as challenges, strive for success, and seek positive outcomes, whereas individuals with avoidance motivation are characterized by a fear of failure and a tendency to avoid punishment or adverse outcomes (Elliot and Covington, 2001; Elliot and Thrash, 2002).

Second, they have different predictive effects. Approach motivation is typically related to adaptive outcomes such as performance attainment and pride (Elliot et al., 2006), whereas avoidance motivation has been found to be associated with maladaptive outcomes, such as poor academic performance (Harackiewicz et al., 2008) and several psychological disorders (Elliot et al., 2006; Lochbaum et al., 2017).

Considering the significant distinction between approach motivation and avoidance motivation (Elliot and Covington, 2001), approach motivation and avoidance motivation may play differential moderating roles in the current mediation effect. Specifically, approach motivation may combine with self-control to reduce the risk of smartphone addiction. When self-control was low, approach motivation may highlight the long-term goals and facilitate a more rational use of smartphones, which usually satisfies individuals with small, immediately available rewards. Therefore, when an individual’s approach motivation is high, the protective effect of self-control on smartphone addiction might be strengthened. When self-control is high, an individual may effectively control smartphone use regardless of the level of approach motivation.

In contrast, individuals with avoidance motivation tend to develop a susceptibility to a “helpless” pattern of responses in achievement settings (Elliot and Covington, 2001). For example, they tend to regard a task as a threat, elicit threat appraisal, and use avoidance-based processes such as strategic withdrawal of effort and procrastination (Rothblum, 1990). Therefore, avoidance motivation may not moderate the indirect effect between self-control and college students’ smartphone addiction. Recent evidence from Chinese adolescents indicates that psychologically controlling parenting increases the risk of mobile phone addiction (Feng et al., 2025), suggesting that such parenting may reinforce maladaptive coping tendencies associated with avoidance motivation. This pattern may be particularly salient in Chinese cultural contexts characterized by high parental expectations and psychologically controlling parenting practices (Chao and Tseng, 2002). In sum, approach motivation may moderate the indirect effect of self-control on smartphone addiction, whereas avoidance motivation may not moderate the indirect effect of self-control on smartphone addiction. Taken together, the person–environment interaction perspective suggests that parental psychological control functions as an environmental risk factor, self-control serves as an individual regulatory resource, and achievement motivation represents an important motivational characteristic that may shape the extent to which self-control influences smartphone addiction. Specifically, the hypotheses are as follows:

*Hypothesis 3*: Approach motivation moderates the mediation effect of self-control on the relationship between parental psychological control and college students’ smartphone addiction.

*Hypothesis 4*: Avoidance motivation is not expected to moderate the mediation effect of self-control on the relationship between parental psychological control and college students’ smartphone addiction.

### 1.4 The present study

**Figure 1:**
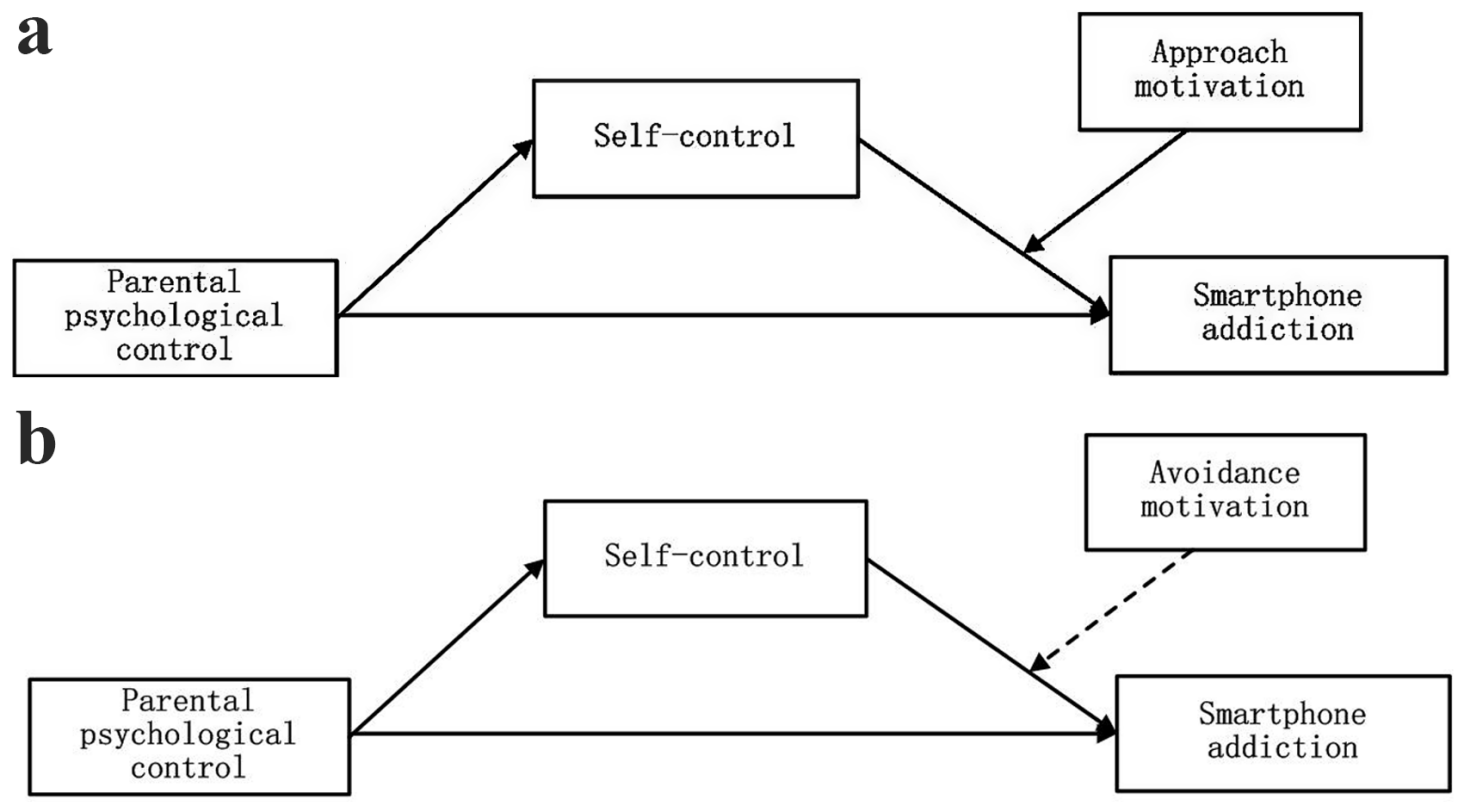
**(a)** Proposed moderated mediation analysis of self-control on the relationship between parental psychological control and college students’ smartphone addiction conditioned by approach motivation. **(b)** Proposed moderated mediation analysis of self-control on the relationship between parental psychological control and college students’ smartphone addiction would not be conditioned by avoidance motivation.

The present study first examined whether self-control plays a mediating role in the relationship between parental psychological control and college students’ smartphone addiction. Second, the present study examined the moderating role of achievement motivation in the indirect pathway. Based on these two research questions, a moderated mediation model can be constructed to examine the influence of parental psychological control on smartphone addiction (see Figures 1a,b).

## 2 Materials and methods

### 2.1 Participants

Following approval from the Research Ethics Committee at the first author’s institution, this study was conducted in accordance with the Declaration of Helsinki, and informed consent was obtained from all participants. A total of 750 undergraduate students were recruited from two universities in Northwest China. The majority of the sample (95%) consisted of first-year students. Among the 730 college students, 402 were female students (*M_age_* = 19.22 years, *SD* = 0.79, range = 18–22 years). Thirty invalid questionnaires due to incomplete data were excluded, resulting in an effective recovery rate of 96.1%. The survey was completed in approximately 20 min. An index of participants’ socioeconomic status (SES) was computed as a composite score following the procedure described by Cohen et al. (2006). Father’s educational level (*M* = 3.58, *SD* = 1.09), mother’s educational level (*M* = 3.38, *SD* = 1.16), and household income (*M* = 2.13, *SD* = 1.02) were standardized based on the full sample, and the mean of the three standardized indicators was used as the continuous composite SES score.

### 2.2 Procedure

The present study was approved by the first author’s institution. We distributed informed consent forms to first-year college students and invited them to participate in the study. College students visited the research laboratory at the scheduled time and were informed of their right to withdraw from the study; however, none chose to do so. They completed questionnaires in an experiment room. The participants received a gift as compensation for their participation.

### 2.3 Measures

#### 2.3.1 Smartphone addiction

The Mobile Phone Addiction Index (MPAI; Leung, 2008) was used to assess smartphone addiction. Given that all the mobile phones used by the participants were smartphones, the MPAI was considered an appropriate measure of smartphone addiction in the present study. It contains four dimensions: inability to control cravings (7 items; e.g., “You have been told that you spend too much time on your mobile phone”), anxiety and feeling lost (5 items; e.g., “You find it difficult to switch off your mobile phone”), withdrawal and escape (3 items; e.g., “You have used your mobile phone to talk to others when you were feeling isolated”), and productivity loss (2 items; e.g., “Your productivity has decreased as a direct result of the time you spend on the mobile phone”). Participants rated each item from 1 (strongly disagree) to 5 (strongly agree), with higher scores indicating higher levels of smartphone addiction. The MPAI has been used in Chinese adolescents and young adults with good reliability and validity (Leung, 2008; Lian et al., 2016; Liu and Wang, 2011). The internal consistency reliability (Cronbach’s *α*) was 0.86 in the present study.

#### 2.3.2 Parental psychological control

Parental psychological control was assessed using a 18-item questionnaire developed by Wang et al. (2007). It contains three dimensions: guilt induction (10 items; e.g., “My parents tell me that I should feel guilty when I do not meet their expectations.”), love withdrawal (5 items; e.g., “My parents act cold and unfriendly if I do something they do not like.”), and authority assertion (3 items; e.g., “My parents tell me that what they want me to do is the best for me and I should not question it.”). The higher the sum score of each dimension, the higher the frequency of perceiving parental psychological control. The internal consistency reliability (Cronbach’s *α*) was 0.819 in the present study.

#### 2.3.3 Self-control

The Chinese version of the Brief Self-Control Scale (BSCS) was used to assess self-control (Tangney et al., 2004; Tan and Guo, 2008). BSCS contains 19 items with five dimensions: self-discipline (6 items), an inclination toward deliberate/non-impulsive action (4 items), an inclination toward healthy habits (3 items), an inclination toward self-regulation in service of work or study (3 items), and reliability (3 items). Participants rated the items from 1 (not at all) to 5 (very much). After reverse-scoring the items, higher scores indicated higher self-control. The internal consistency reliability (Cronbach’s α) was 0.85 in the present study.

#### 2.3.4 Achievement motivation

Achievement motivation was measured using the Achievement Motivation Scale revised by Ye and Hagtvet (1992). The scale contains 15 odd-numbered questions that measure approach motivation and 15 even-numbered questions that measure avoidance motivation. For each item, participants responded using a 5-point scale, ranging from 1 (do not agree at all) to 5 (very strongly agree). The Cronbach’s α coefficients for internal consistency reliability were 0.80 and 0.78 in the present study.

#### 2.3.5 Control variables

Control variables included sex (female vs. male) and SES. Participants reported their age and sex using a demographic questionnaire. SES was computed as described above.

### 2.4 Analysis process

First, SPSS (version 24.0) was used to calculate *P*earson’s correlations among the study variables. Second, models 4 and 15 of the SPSS macro PROCESS (Hayes, 2013) were used to test the mediation pathway and the moderated mediation model.

## 3 Results

### 3.1 Common method biases

To avoid common method biases, this study collected anonymous responses and reverse-scored some items. This study tested for common method biases using Harman’s single-factor test. Seven factors with eigenvalues greater than 1 were extracted, and the first factor explained 29.36% of the variance. The results showed that there is no serious common method bias in this study (Zhou and Long, 2004).

### 3.2 Descriptive statistics

The means and SDs of all variables and the correlations between these variables are presented in Table 1.

**Table 1:** Descriptive statistics and correlations.

Variables	1	2	3	4	5	6	7
1 Sex	1						
2 SES	0.09^*^	1					
3 Parental psychological control	0.22^***^	0.07	1				
4 Self-control	0.04	0.09^*^	−0.20^**^	1			
5 Approach motivation	0.05	−0.02	0.03	0.06	1		
6 Avoidance motivation	−0.03	0.01	0.12^**^	−0.33^**^	0.28^**^	1	
7 Smartphone addiction	0.08*	−0.001	0.39^**^	−0.40^**^	−0.12	0.33^**^	1
*M*	−	−	44.87	64.45	47.49	45.76	45.02
*SD*	−	−	11.68	9.20	9.03	9.69	12.36

*p < 0.05, **p < 0.01, and ***p < 0.001.

### 3.3 Testing direct relations

The results suggested that parental psychological control was positively associated with smartphone addiction (*β* = 0.39, *p <* 0.001; 95% CI [0.26, 0.42]).

### 3.4 Testing the mediation model

As shown in Table 2, after controlling for sex and SES, parental psychological control was negatively associated with self-control (*β* = −0.22, *p* < 0.001), and self-control was negatively associated with smartphone addiction (*β* = −0.34, *p* < 0.001). Moreover, when self-control was included, parental psychological control was still positively associated with smartphone addiction (*β* = 0.32, *p* < 0.001). Finally, based on 5,000 bootstrap samples, the indirect effect of parental psychological control on smartphone addiction through self-control was 0.07 (95% CI [0.04, 0.11]). The 95% CI did not contain zero, indicating that self-control partially mediated the association between parental psychological control and smartphone addiction.

**Table 2:** Mediation analysis.

Outcome variable	Predictor variable	*R* ^2^	*F*	*β*	*SE*	LLCI	ULCI	*t*
Self-control	Sex	0.05	13.98^***^	0.08	0.04	0.01	0.15	2.07^*^
	SES			0.10	0.04	0.001	0.03	2.78^**^
	Parental psychological control			−0.22	0.04	−0.29	−0.15	−6.05^***^
Smartphone addiction	Sex	0.26	64.65^***^	0.02	0.03	−0.05	0.08	0.53
	SES			0.01	0.03	−0.06	0.07	0.24
	Parental psychological control			0.32	0.03	0.26	0.39	10.07^***^
	Self-control			−0.34	0.03	−0.40	−0.27	−10.86^***^

*p < 0.05, **p < 0.01, and ***p < 0.001. SES, socioeconomic status.

### 3.5 Testing the moderated mediation model

**Figure 2:**
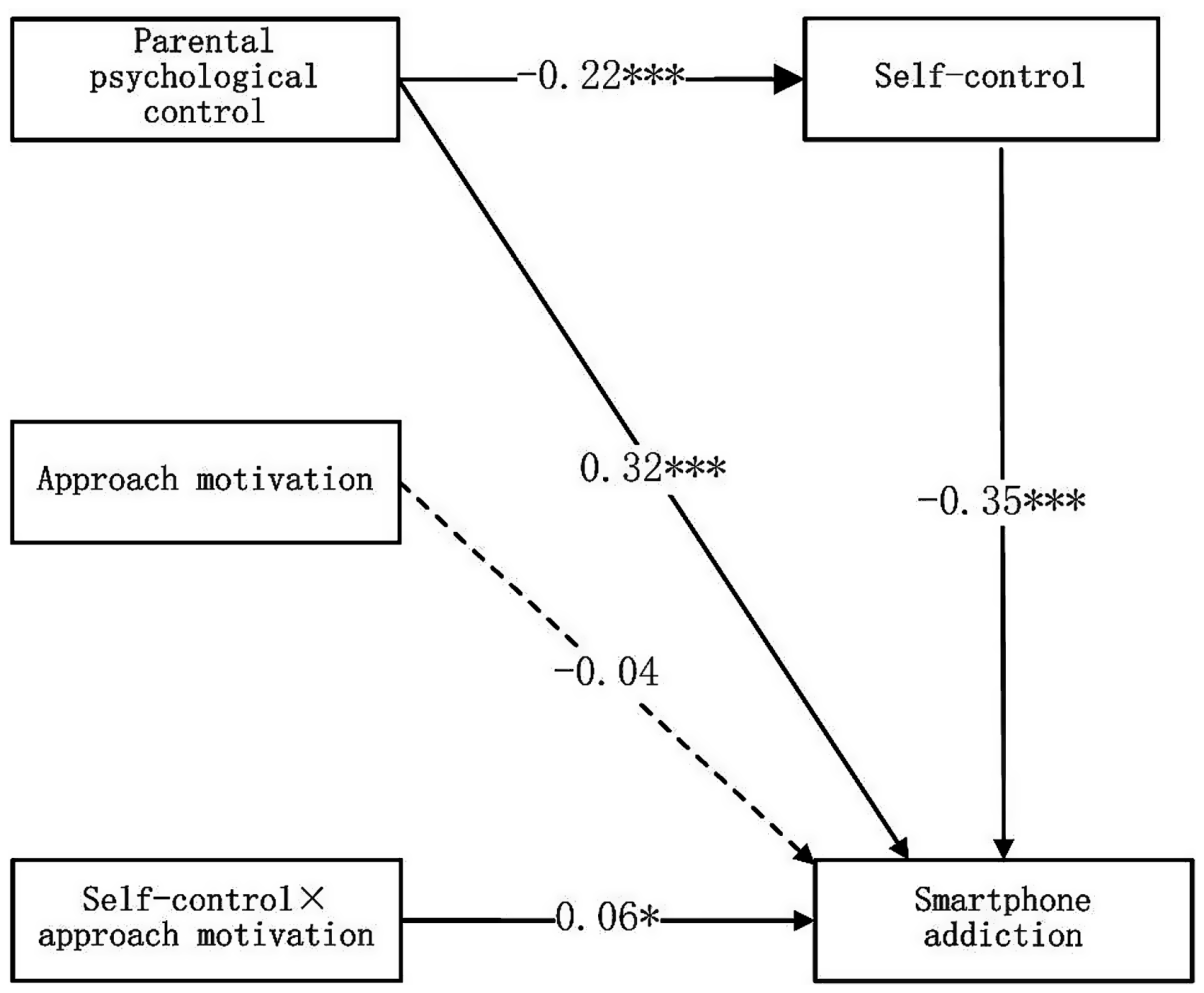
Moderated mediation analysis of self-control on the relationship between parental psychological control and college students’ smartphone addiction conditioned by approach motivation.

As shown in Table 3 and Figure 2, after controlling for sex and SES, self-control was negatively associated with smartphone addiction (*β* = −0.35, *p* < 0.001), while the interaction between self-control and approach motivation was negatively associated with smartphone addiction (*β* = 0.06, *p* < 0.05). Thus, approach motivation was observed to moderate the relationship between self-control and smartphone addiction.

**Table 3:** The moderated mediation model (moderated by approach motivation).

Predictor	*β*	SE	LLCI	ULCI	*t*
Sex	0.02	0.03	−0.05	0.08	0.53
SES	0.005	0.03	−0.06	0.07	0.14
Parental psychological control	0.32	0.03	0.26	0.39	9.66^***^
Self-control	−0.35	0.03	−0.42	−0.29	−10.60^***^
Approach motivation	−0.04	0.03	−0.10	0.03	−1.04
Self-control × approach motivation	0.06	0.03	0.01	0.12	2.40^*^

*p < 0.05, **p < 0.01, and ***p < 0.001. SES, socioeconomic status.

**Figure 3:**
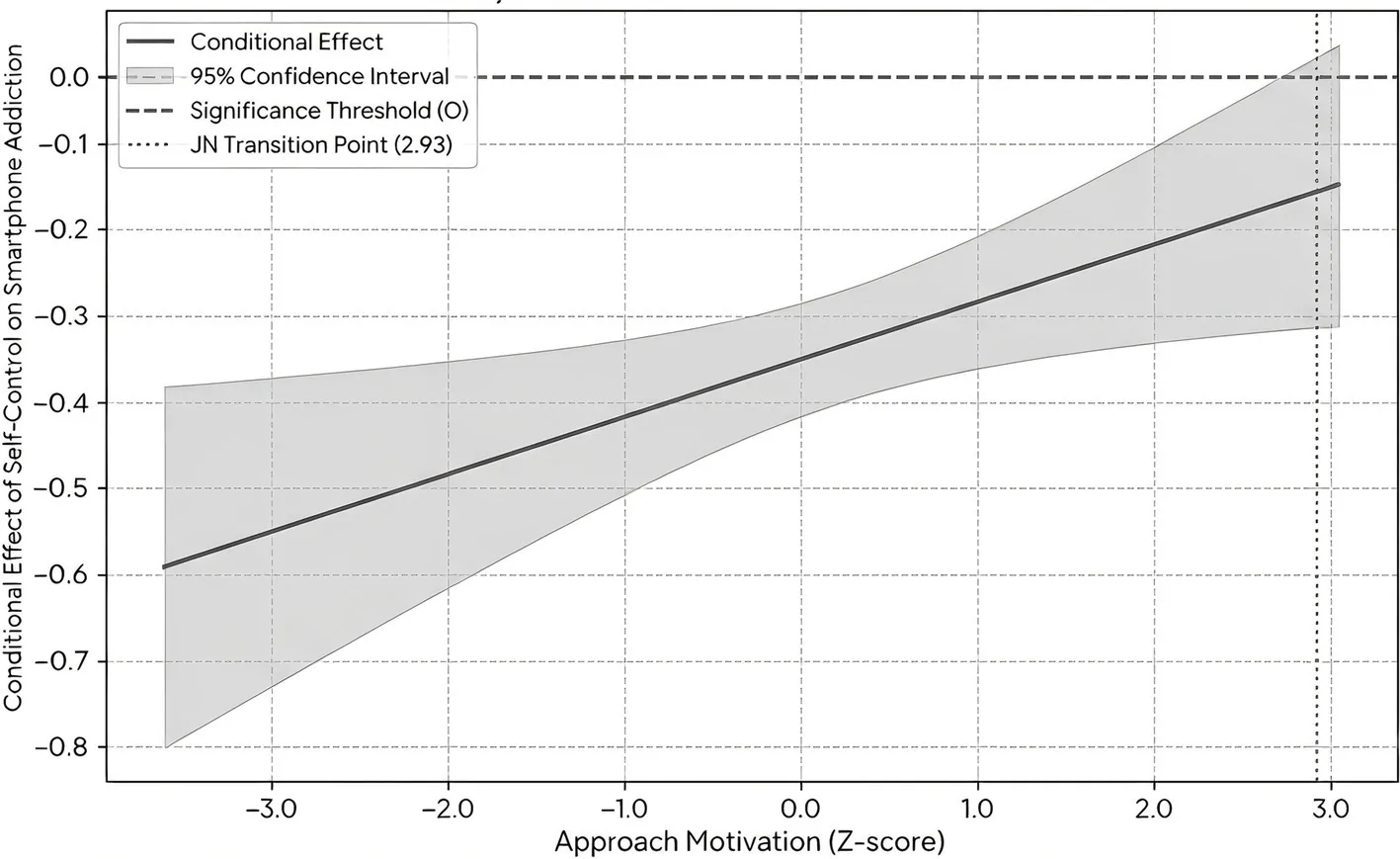
Johnson–Neyman plot: conditional effect of focal predictor.

**Figure 4:**
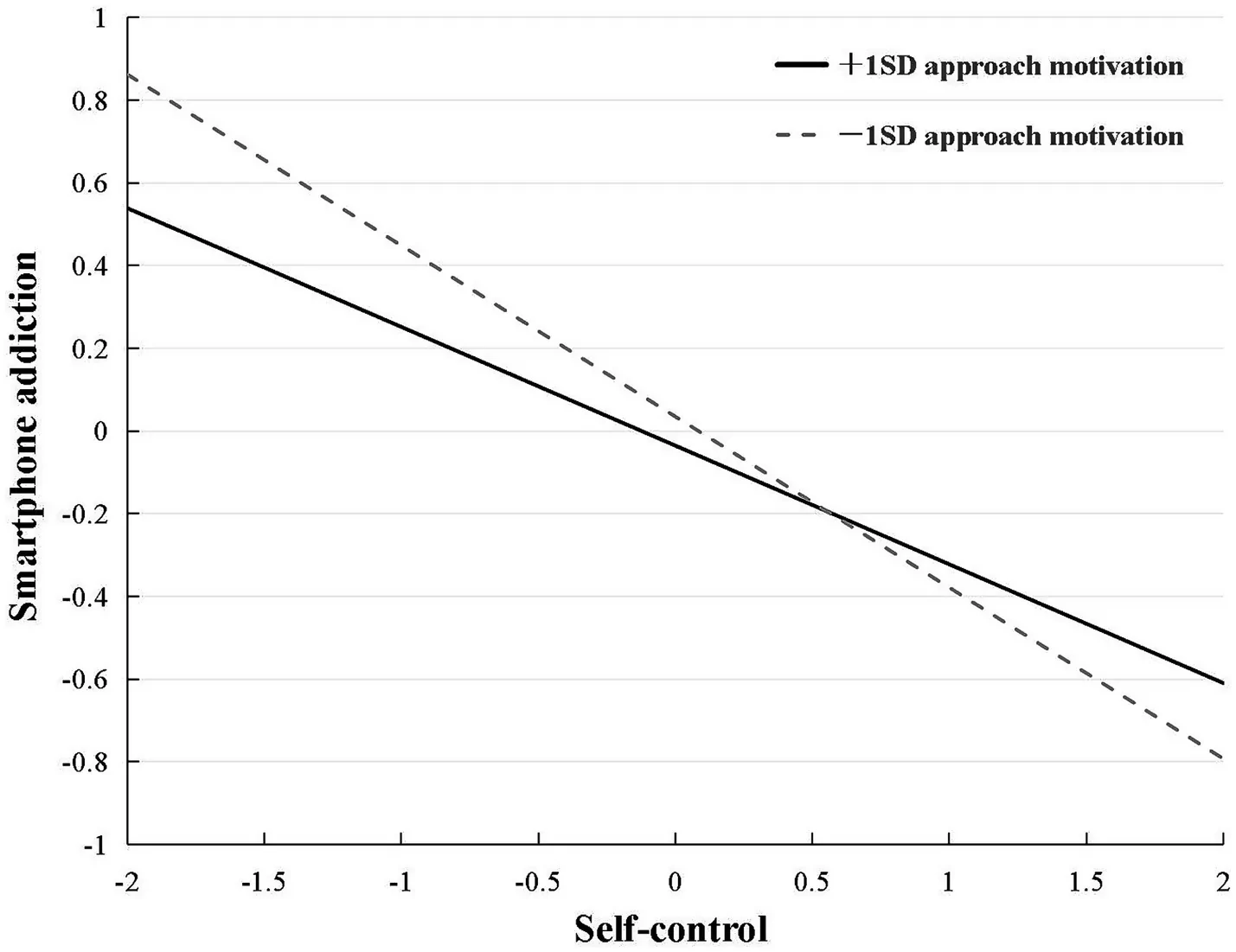
Moderating effect of approach motivation on the indirect pathway between self-control and smartphone addiction.

To further probe this moderation effect, we conducted a Johnson–Neyman (J–N) analysis (Figure 3). The analysis revealed that the conditional effect of self-control on smartphone addiction remained consistently negative across the observed range of approach motivation. The J–N transition point was identified at 2.93 (a Z score of approach motivation), indicating that the effect remained significant across nearly the entire observed range of approach motivation. Simple slope analyses (Figure 4) further showed that the negative association between self-control and smartphone addiction remained significant at both low and high levels of approach motivation. Bootstrap analyses further indicated that the conditional indirect effect of parental psychological control on smartphone addiction through self-control remained significant at low (effect = 0.09, 95% CI = [0.05, 0.14]), mean (effect = 0.08, 95% CI = [0.04, 0.12]), and high (effect = 0.06, 95% CI = [0.03, 0.10]) levels of approach motivation. None of the bootstrap confidence intervals included zero, indicating that the indirect effect remained significant across different levels of approach motivation, although its magnitude gradually decreased as approach motivation increased.

As shown in Table 4, avoidance motivation did not moderate the indirect effects of parental psychological control on smartphone addiction via self-control (*β* = −0.001, *ps* > 0.05).

**Table 4:** The moderated mediation model (moderated by avoidance motivation).

Predictor	*β*	SE	LLCI	ULCI	*t*
Sex	0.02	0.03	−0.04	0.09	0.75
SES	−0.001	0.03	−0.06	0.06	−0.02
Parental psychological control	0.32	0.03	0.25	0.38	9.89^***^
Self-control	−0.28	0.03	−0.35	−0.21	−8.37^***^
Avoidance motivation	0.20	0.04	0.14	0.27	5.76^***^
Self-control × avoidance motivation	−0.001	0.03	−0.07	0.07	−0.04

*p < 0.05, **p < 0.01, and ***p < 0.001. SES, socioeconomic status.

## 4 Discussion

By testing two moderated mediation models, the present study examined the mediating role of self-control in the relationsip between parental psychological control and college students’ smartphone addiction and whether this mediating relationship was moderated by college students’ approach motivation and avoidance motivation. The present study found that beyond the direct effect, parental psychological control was associated with college students’ smartphone addiction through the partial mediation role of self-control. Approach motivation moderated the mediation pathway between self-control and college students’ smartphone addiction, while avoidance motivation did not moderate the mediation pathway. The finding extends previous literature by revealing that individual factors combined with family environmental factors contribute to college students’ smartphone addiction.

The present study found that parental psychological control was positively related to college students’ smartphone addiction. In line with previous studies, the finding further supports the notion that parents continue to exert influences on their teenagers’ problem functioning after they enter college (Abar and Turrisi, 2008). The findings of the present study are consistent with previous findings that parental psychological control was associated with Internet addiction and drug and alcohol dependence (Torresani et al., 2000). When college students cannot obtain sufficient support from their parents, they may seek reassurance and alleviate distress in the virtual world.

Furthermore, parental psychological control exerted not only a direct effect but also an indirect effect on smartphone addiction through self-control. Specifically, parental psychological control could influence college students’ self-control, which, in turn, increased their risk of smartphone addiction. The result extends previous findings that parental psychological control may indirectly contribute to addictive behaviors through its association with self-control (Cecil et al., 2012). Parents who excessively interfere with or control their children may hinder the development of college students’ self-control (Crosswhite and Kerpelman, 2012; Li et al., 2013). Consequently, those college students may not resist the temptation of the virtual world. They may increasingly use their smartphones habitually rather than in a controlled manner and gradually exhibit smartphone addiction. On the other hand, less psychological control may promote their children’s socialization (Huang et al., 2010) and facilitate the development of self-control, which, in turn, may reduce the likelihood of smartphone addiction.

Furthermore, the present study found that approach motivation moderated the indirect pathway from parental psychological control to college students’ smartphone addiction through self-control. Specifically, a high level of self-control was related to lower levels of smartphone addiction, regardless of the level of approach motivation. The association between low self-control and smartphone addiction depended on the level of approach motivation. When self-control was deficient, individuals with higher approach motivation showed lower levels of smartphone addiction than those with lower approach motivation. Consistent with the dual-motive model (Fujita, 2011), approach motivation may serve as an influential factor that compensates for low self-control. College students with high approach motivation are motivated by positive outcomes and pursue success as their goal (Elliot and Thrash, 2002). During the process of achieving this goal, individuals are more likely to exert greater effort and persistence. Thus, when encountering salient proximal temptations, they are more likely to choose their distal reward and long-term goals, thereby increasing the likelihood of successful self-control. They could more efficiently plan and monitor their current behaviors (Fishbach et al., 2003) and also be less likely to overuse smartphones. In addition, these findings also provide empirical support for the buffering role model (Cohen, 2003). Specifically, approach motivation could buffer the detrimental effect of negative parental psychological control on self-control and subsequent smartphone addiction. It is worth noting that the buffering role of approach motivation, like that of other individual characteristics, is limited based on our current findings. Approach motivation may not be sufficient to protect college students with poor self-control from smartphone addiction in the face of negative parenting.

As expected, the present study did not find a significant moderating effect on the indirect pathway from parental psychological control and self-control to college students’ smartphone addiction. However, the present study found a significant positive association between avoidance motivation and smartphone addiction. Compared to the significant moderation effect of approach motivation, this result highlights the distinct functional roles of these two motivational orientations within the hierarchical model of achievement motivation (Elliot and Church, 1997).

The reason for this difference lies in their distinct ways of influencing behavior. Approach motivation is driven by the desire for success, which encourages goal-directed effort and provides a “buffer” that helps students overcome low self-control. Conversely, avoidance motivation is rooted in the fear of failure and the desire to avoid incompetence (Elliot and Church, 1997; Elliot et al., 2006). This “threat focus” creates a persistent state of anxiety rather than providing the energy needed for self-regulation. These findings suggest that approach and avoidance motivation should not be regarded as opposite ends of a single continuum but as two functionally distinct motivational systems.

Two factors may explain why avoidance motivation does not moderate the self-control pathway. First, the anxiety linked to avoidance motivation tends to consume cognitive resources. Instead of helping individuals monitor their smartphone use, avoidance motivation keeps them preoccupied with potential failure, leaving limited mental resources to regulate their behavior, regardless of their self-control level. Second, avoidance motivation is closely linked to avoidant coping strategies, such as procrastination and effort withdrawal. As individuals driven by avoidance motivation often consider the smartphone an “escape tool” when confronting difficulties or stress, this motivation directly increases the tendency toward self-sabotage and excessive usage (Lochbaum et al., 2017). Consequently, avoidance motivation acts as a direct risk factor that drives addiction autonomously rather than interacting with the individual’s executive self-control to mitigate the risk.

### 4.1 Limitations

Several limitations of the present study should be noted. First, although the moderated mediation model used in this study was established on both a theoretical and an empirical basis, the cross-sectional design cannot establish causal relationships among variables. Subsequent longitudinal studies should further clarify the causal relationships investigated in the present study. Second, this study collected self-control data via a self-reported questionnaire; future studies can consider using experiments to assess it.

### 4.2 Contributions and practical implications

This study provides a clearer picture of how parental psychological control, self-control, and achievement motivation work together to affect smartphone addiction, using a person–environment interaction perspective. Previous studies have often looked at these factors separately; however, our study connects them to show how family environment and personal traits jointly influence behavior. We found that parental psychological control impairs a student’s ability to regulate their smartphone use by damaging their internal self-control. Importantly, few previous studies have simultaneously examined mediation (self-control) and two distinct moderators (approach vs. avoidance motivation) within one integrated model. Our findings extend the dual-motive model (Fujita, 2011) by showing that approach motivation helps students stay focused on goals and reduces addiction risk, while avoidance motivation acts as a direct risk factor.

These findings also offer practical guidance for prevention and intervention. First, parents should be encouraged to replace psychological control with autonomy-supportive parenting, even after children enter college. Reducing guilt induction and love withdrawal can help preserve young adults’ self-regulation, which is critical for preventing addictive behaviors. Second, the mediating role of self-control suggests that universities should include self-control training in mental health curricula. Counselors can offer structured workshops on emotion regulation and impulse management (Pesce et al., 2020), helping students build skills to resist excessive smartphone use. Furthermore, incorporating physical activity into university-based intervention programs may also be beneficial (Yang et al., 2021) as acute aerobic exercise has been shown to reduce smartphone craving among college students with mobile phone dependency (Yang et al., 2022). Third, for college students themselves, interventions should address motivational shifts. Given that Chinese college students’ achievement motivation has declined over the past two decades, whereas avoidance motivation has increased (Yang et al., 2026), students with low approach motivation may particularly benefit from goal-setting programs. These programs can help them identify meaningful academic or personal goals and reframe smartphone use as either supporting or hindering those goals—thereby reducing its appeal as an avoidant coping strategy.

## 5 Conclusion

The present study elucidated the potential mechanism underlying the relationship between parental psychological control and college students’ smartphone addiction. Our findings suggested that self-control may be a crucial individual factor in the association between parental psychological control and smartphone addiction among college students. Moreover, the moderated mediation model showed that approach motivation moderated the association between self-control and smartphone addiction.

## Data Availability

Due to participant privacy and ethical constraints, the dataset is not publicly available. Requests to access the datasets should be directed to by394272702@163.com.
